# Inhibition of Phosphatidylinositol 3-kinase (PI3K) Signaling Synergistically Potentiates Antitumor Efficacy of Paclitaxel and Overcomes Paclitaxel-Mediated Resistance in Cervical Cancer

**DOI:** 10.3390/ijms20143383

**Published:** 2019-07-10

**Authors:** Jing Jing Liu, Jung Yoon Ho, Hye Won Lee, Min Wha Baik, Oyoung Kim, Youn Jin Choi, Soo Young Hur

**Affiliations:** 1 Department of Gynecology and Obstetrics, Seoul St. Mary’s Hospital, The Catholic University of Korea, Seoul 06591, Korea; 2 Cancer Research Institute, Department of Medical Life Science, and Cancer Evolution Research Center, College of Medicine, The Catholic University of Korea, Seoul 06591, Korea; 3 Department of Health Sciences and Technology, SAIHST, Sungkyunkwan University, Seoul 06591, Korea

**Keywords:** cervical cancer, combination therapy, paclitaxel resistance, cell cycle, invasion, PI3K inhibitor

## Abstract

Acquired paclitaxel (PTX) resistance limits its effectiveness and results in advanced cancer progression. This review investigated whether the inhibition of phosphatidylinositol 3-kinase (PI3K) signaling overcomes paclitaxel resistance in cervical cancer. It was established paclitaxel-resistant cell lines (PTX-R ME180/PTX-R HeLa) and determined the combination index for paclitaxel and PI3K inhibitors (BYL-719/ LY294002) by tetrazolium dye assay. Flow cytometry was used to detect the cell cycle and apoptosis. Migration and invasion were explored by wound healing and transwell assays. Genes related to multiple pathways were assessed by a western blot. It was found that the PI3K pathway was significantly activated in paclitaxel-resistant HeLa and ME180 cells compared to parental cells. PTX + PI3K inhibitor combined therapy showed a synergistic effect by strengthening paclitaxel-induced S and G_2_M arrest in PTX-R cell sublines by the inactivation of cyclin A1, cyclin B1, cyclin E, and Cdc2 expression. Moreover, combination therapy significantly enhanced drug sensitivity and apoptosis through the activation of Bax, and cleavage of poly-(ADP-ribose) polymerase compared with paclitaxel alone. In addition, PI3K inhibition also suppressed tumor migration and invasion by targeting β-catenin and matrix metalloproteinase-2/9. The authors suggest that the combination of a PI3K inhibitor with paclitaxel may enhance antitumor activity through a cascade of PI3K signaling events.

## 1. Introduction

Cervical cancer is the third most common gynecological malignancy and responsible for 10–15% of cancer-related deaths in women [1]. Even with surgery followed by aggressive front-line chemotherapy, which can yield cures in 80% to 90% of women with early stage I and II cervical cancers, the problem of advanced or recurrent cervical cancer failing to respond to conventional treatment remains unresolved [2,3,4].

Paclitaxel (PTX) is a microtubule-stabilizing agent widely employed as a front-line chemotherapeutic agent for cervical cancer (used alone or in combination with other therapeutic agents). It has a response rate of 29–63%, mainly due to acquired chemo-resistance, leading to a dismal prognosis [5,6]. Therefore, overcoming chemo-resistance remains a great challenge in recurrent and advanced cervical cancer treatment. Resistance is a complex phenomenon associated with multiple mechanisms, including deficient apoptosis [7], the expulsion of cancer drugs by cell membrane transporters [8], and the activation of multiple signaling pathways [9]. To date, the mechanisms involved in PTX resistance have not been fully elucidated. The discovery of new resistance reversal agents with low toxicity that target resistance biomarkers is a major goal of cancer biologists and pharmacists.

Recent advances in molecular biology have led to a new concept of medical treatment other than conventional chemotherapy [10]. Targeted therapy is expected to be successful in managing advanced, persistent or recurrent cervical cancers. Excessive activation of the phosphatidylinositol 3-kinase (PI3K) pathway is considered to play an important role in the regulation of the cell cycle [11], proliferation [12], differentiation [13], survival and metabolism [14], and the processes of tumor development and metastasis [15] in multiple tumors. Based on their different structures, specific substrates, and different modes of regulation, PI3K genes are divided into three classes (I, II, and III) [16]. Activating mutations or a copy number gain of *PIK3CA* at 3q26 encoding PI3K-p110α are the most significantly consistent chromosomal alterations found in primary cervical cancer, highlighting its important role in the progression of dysplastic uterine cervical cells to invasive cancer [17]. The hotspot mutations, E542K and E545K/Q, are located mostly at the interface between p110α helical (Glu542, Glu545) and p85α nSH2 domains [18], leading to increased enzymatic activity that can initiate PI3K proto-oncogene functions [19]. It is suggested that the class I PI3K gene, *PIK3CA*, stands out owing to its essential role in the PI3K/AKT signaling pathway and cervical cancer.

Previous studies have described how PI3K inhibitors alone or in combination with other anti-cancer agents can replace conventional chemotherapeutic agents when cancer resistance is induced [20]. BYL719 is a selective PI3Kα inhibitor [21] that shows increased sensitivity, especially in preclinical models harboring *PIK3CA* mutations. It induced fewer toxicities and had a more favorable safety profile compared to a pan class I PI3K inhibitor [22]. LY294002, one of the earliest synthetic PI3K inhibitors, is still widely used in diverse signal transduction processes involving the PI3K pathway, despite its limitation in clinical trials due to unfavorable pharmacokinetic properties and high toxicity [23]. Moreover, studies have yielded evidence that PI3K signaling is associated with paclitaxel sensitivity in various malignancies [24]. Sensitivity to paclitaxel increased via the PI3K pathway when paclitaxel-resistant prostate cancer cells were treated with LY294002 [25]. In addition, blockade of the PI3K pathway inhibited paclitaxel-resistant ovarian cancer cell proliferation and migration, and reversed the sensitivity of these cellular processes to paclitaxel [26]. Clinical data shows that combining the PI3K inhibitor, BYL-719, with a taxane was well tolerated, indicating this may be a possible approach to treating advanced solid tumors [27]. It continues to be investigated in a large cohort (NCT02379247). However, the relationship between PI3K and paclitaxel resistance has not yet been fully elucidated in cervical cancer. In this study, the authors attempted to identify whether inhibiting the PI3K signaling pathway would yield enhanced paclitaxel sensitivity in paclitaxel-resistant cervical cancer.

## 2. Results

### 2.1. Genetic Patterns of PIK3CA Aberrations Exhibit Oncogenic Functions in Advanced Stage Cervical Cancer

**Figure 1:**
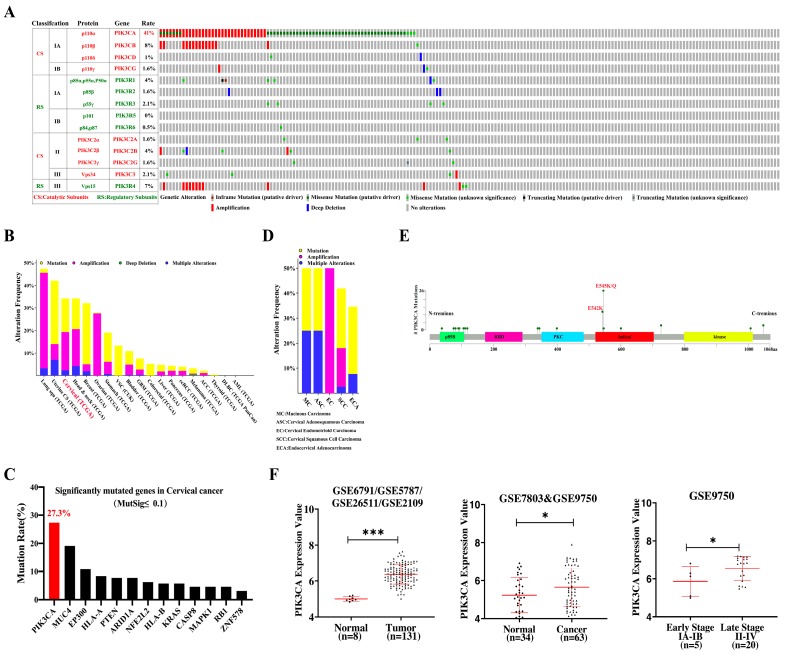
High frequency alteration events of the *PIK3CA* gene in cervical cancer based on TCGA and GEO databases. (**A**) Overview of phosphatidylinositol 3-kinase (PI3K) isoforms. In cervical cancer *PIK3CA*, the gene encoding p110α alterations, occupied the highest level among the whole family of genes. The PI3K family consists of eight isoforms that are mainly grouped into three classes (I, II, III) based on protein homology and enzyme affinity for specific phosphoinositide substrates. The class I isoforms are further subdivided into class IA and class IB isoforms, referring to their mechanism of activation, with each isoform composed retrieved d of a regulatory and catalytic subunit. (**B**) Cross-cancer alteration summary for phosphoinositide-3-kinase, catalytic, alpha (*PIK3CA*) polypeptide according to The Cancer Genome Atlas (TCGA) database (retrieved from the cBioPortal for Cancer Genomics). Mutations are represented in yellow, deletions in green, amplification in rose red, and multiple alterations in violet blue. Abbreviations: Lung squ: lung squamous cell carcinoma; DLBC: lymphoid neoplasm diffuse large B-cell lymphoma; AML: acute myeloid leukemia; GBM: glioblastoma multiforme; uterine CS: uterine carcinosarcoma; CcRCC: kidney renal clear cell carcinoma; ACC: adrenocortical carcinoma; VSC(CUK): squamous cell carcinoma of the vulva (CUK, Exp Mol Med 2018).(**C**) *PIK3CA* is one of the most frequently mutated genes in cervical cancer (cBioPortal). (**D**) *PIK3CA* alterations in different types of cervical cancer based on the TCGA database (retrieved from cBioPortal). (**E**) Representative graphical summary of *PIK3CA* mutations mapped across the gene in 191 cervical cancer TCGA samples (from cBioPortal, which provides visualization, analysis and downloads of large-scale cancer genomic datasets, http://www.cbioportal.org/). Green circles represent missense mutations. A “hotspot” mutation, mapped to two sites, E545 and E542, in the helical domain (exon 9) of p110α. (**F**) Increased mRNA expression of *PIK3CA* in cervical cancer shows a significant positive relationship with the stage of advanced cervical cancer, compared to early stage cancer, based on two platforms consisting of multiple Gene Expression Omnibus (GEO) series datasets. *p < 0.05, ***p < 0.001.

Class I PI3K genes determine the activity of PI3K/AKT signaling and are the most frequently occurring genetic alterations (rate: 41%) of the whole PI3K family in cervical cancer (Figure 1A). Based on a The Cancer Genome Atlas (TCGA) database, significant *PIK3CA* alterations were analyzed, such as mutations, amplification and deletions, which revealed a relatively high frequency of alterations across multiple cancers, including cervical cancer (Figure 1B). Moreover, it was found that the *PI3KCA* mutation rate was 27.3%, ranking first place in the top 10 mutation gene list identified by MutSig with q ≤ 0.1 (Figure 1C). Additionally, data generated from cBioPortal (Figure 1D) revealed that the *PIK3CA* mutation showed a high alteration rate in squamous cell carcinoma, adenocarcinoma, adenosquamous carcinoma and mucinous adenocarcinoma compared to endometrioid carcinoma. Specific mutations within *PIK3CA* that cluster in hotspots located in exon 9 (E542/545K) (Figure 1E) were demonstrated to enhance the activation of PI3K/AKT signaling, and were involved in carcinogenesis as well as chemotherapy resistance [28]. Further, the authors downloaded and analyzed raw CEL files from Gene Expression Omnibus (GEO) database and found that the *PIK3CA* mRNA expression level from the GPL570 platform (GSE2109/GSE6791/GSE5787/GSE26511) was significantly upregulated in 131 cancer cases compared to eight normal cases (Figure 1F, *p* < 0.001). A similar result was also found in 63 cervical cancer tissues versus 34 normal tissues from a GPL96 (GSE7803/GSE9750) platform (Figure 1F, *p* < 0.05). Additional analysis for *PIK3CA* in different stages of cervical cancer obtained from an available clinical information dataset, GSE9750, indicated that high *PIK3CA* amplification was significantly associated with advanced stages in cervical cancer (Figure 1F, *p* < 0.05). Taken together, *PIK3CA* alterations, including mutations and amplification, may be a good marker to predict tumor progression and chemotherapy resistance, possibly providing a novel concept in the treatment of advanced cervical cancer.

### 2.2. Involvement of PIK3CA Alterations in the Process of Paclitaxel Resistance and its Sensitivity to BYL-719 and LY294002 in Cervical Cancer Cell Lines

**Figure 2:**
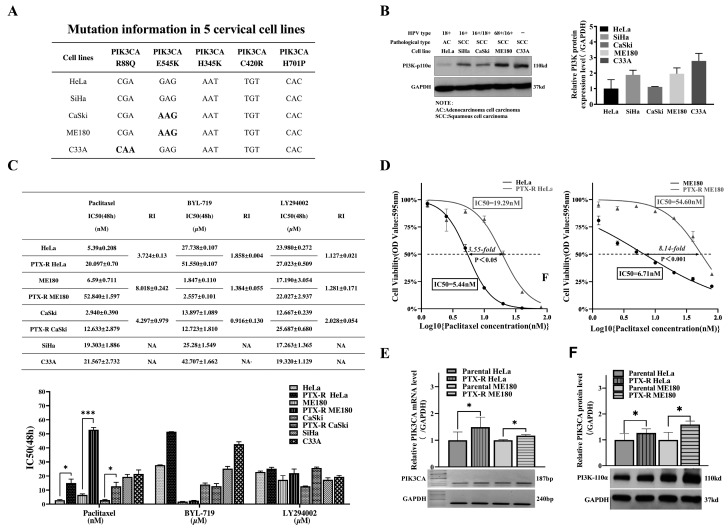
*PIK3CA* mutational status, expression in cervical cell lines, and its alterations and chemo-sensitivity to paclitaxel and BYL-719 or LY294002 after the induction of paclitaxel-resistant cells. (**A**) Hotspot mutation analysis for *PIK3CA* is summarized in a panel of human cervical cancer cell lines based on The Cancer Cell Line Encyclopedia (CCLE) project. (**B**) The protein expression of *PIK3CA* in five common cervical cancer cell lines, with or without high-risk human papilloma virus (HPV)-type infections, that were initiated from different pathological types were studied by western blot assay. The representative bands are shown on the left. (**C**) The half-maximal inhibitory concentration (IC50) values for paclitaxel, BYL-719, and LY294002 were calculated for five cervical cancer cell lines as well as three paired paclitaxel-resistant cell lines after 48 h. *p < 0.05, **p < 0.01. ***p < 0.01. (**D**) Cell viability for paclitaxel chemosensitivity was tested and a resistance index (RI) was calculated in cells with acquired resistance (paclitaxel-resistant [PTX-R] HeLa, PTX-R ME180) after 48 h using a tetrazolium dye(MTT) assay. The two PTX-R cell lines were more resistant to paclitaxel compared to the parental cell lines (HeLa, ME180). (**E**,**F**) Alterations in *PIK3CA* mRNA and protein expression were investigated after the establishment of resistant HeLa or ME180 cells. Semi-quantitative reverse transcriptase (sqRT–PCR) analysis (E) and western blot assays (F) confirmed that the level of *PIK3CA* was significantly up-regulated in paclitaxel-resistant cells compared to parental cells. *p < 0.05.

The mutation data sheet downloaded from the CCLE website (Figure 2A) was first summarized and western blots were performed to assess the difference in expression of *PIK3CA* in four human papilloma virus (HPV)-positive types, including five cervical cancer cell lines. Our data illustrated that, compared to primary tissue, derived HeLa, ME180 and CaSki cell lines, originally established from metastatic sites of cervical cancer, harbored the *PIK3CA* E545K mutation. The highest *PIK3CA* level was found in the four HPV positive cells, excluding a higher level in C33A with an R88Q mutation (Figure 2B). Next, the inhibitory concentration IC(50) was measured for paclitaxel in cervical cell lines. The paclitaxel half maximal inhibitory concentration (IC50) in parental HeLa, ME180, CaSki, SiHa, and C33A cell lines was 5.39 ± 0.208, 6.59 ± 0.711, 2.940 ± 0.390, 19.303 ± 1.886, and 21.567 ± 2.732 nM, respectively (Figure 2C). After 7 months with a low-dose paclitaxel escalation from 1 nM, HeLa and ME180 resistant sublines stably exhibited 3.55- and 8.14-fold resistance to paclitaxel compared to parental cells and were stably resistant to paclitaxel under 20 nM (Figure 2D), while CaSki-resistant sublines gained a 4.54-fold resistance to paclitaxel (Figure 2C). The resistance index (RI) was in the range of previously reported RI values for cells established from patients with clinical cancer [29]. The samples from HPV-positive HeLa and ME180 cell lines and resistant sublines showing the highest and lowest *PI3KCA* expression levels were selected and assayed by a western blot and semi-quantitative reverse transcriptase (sqRT)–PCR. The *PIK3CA* mRNA level was found to be significantly upregulated in paclitaxel-resistant (PTX-R) HeLa and PTX-R ME180 cells compared to parental cell lines (*p* < 0.05, Figure 2E,F). This suggests that *PIK3CA* activation is involved in paclitaxel resistance and may thus be a clinically relevant target to overcome chemoresistance and prevent metastasis during paclitaxel chemotherapy in cervical cancer.

Since *PIK3CA* was activated in paclitaxel-resistant cells and cells with *PIK3CA* mutations were reported to show increased sensitivity to PI3K inhibitors [30], the authors examined if the combination of paclitaxel with either BYL-719(BYL) or LY294002(LY) could restore paclitaxel sensitivity in PTX-R HeLa and ME180 cells. A tetrazolium dye (MTT) assay was first used to explore parental and paclitaxel-resistant HeLa, CaSki and ME180 cell lines for sensitivity to the two aforementioned PI3K inhibitors. It was found that parental ME180 cells with *PIK3CA* mutations exhibited greater sensitivity to BYL than *PIK3CA* wild-type cell lines (HeLa and SiHa). CaSki showed a modest sensitivity to BYL, while C33A was insensitive to BYL (IC50 42.707 μM, Figure 2C). In comparison, the IC50 for LY in these five cervical cell lines were at a similar level of between 17.19 to 23.98 μM (Figure 2C). Both the RI of BYL and LY exhibited no more than a two-fold change in PTX-R HeLa and PTX-R ME180 cell lines compared to parental cells. Finally, HeLa, and ME180 cell lines as well as their resistant sublines were selected for further study.

### 2.3. Therapeutic Synergistic Effect of PI3K Inhibitors (BYL-719 and LY294002) and Paclitaxel Can Increase Sensitivity to Paclitaxel Cytotoxicity and Inhibit the PI3K/AKT Pathway in Paclitaxel-Resistant Cells

**Figure 3:**
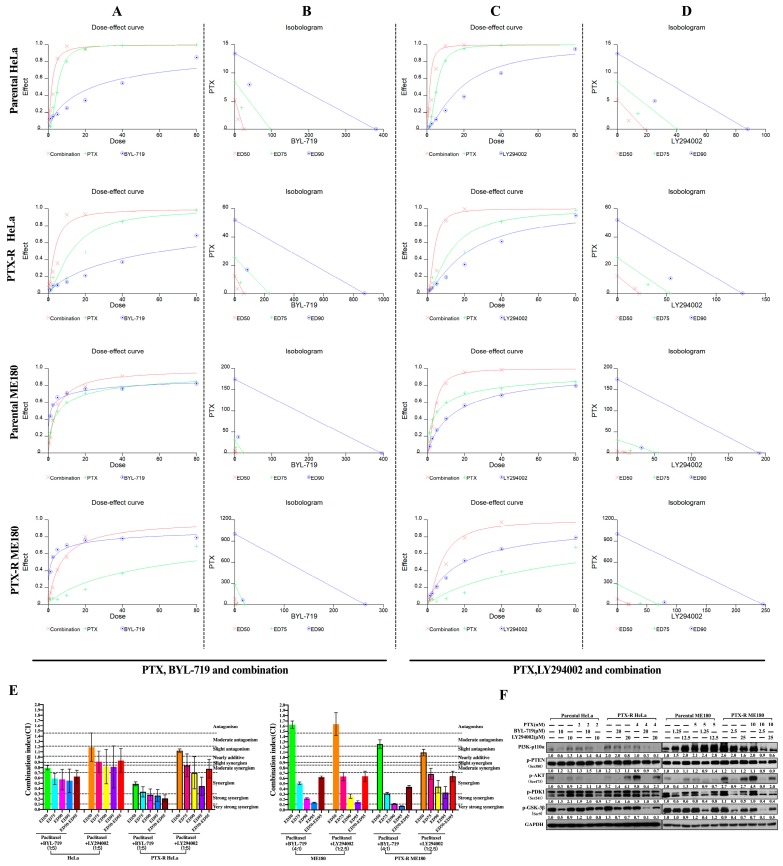
Synergistic interaction of BYL-719 or LY294002 in combination with paclitaxel in the treatment of parental and paclitaxel-resistant HeLa and ME180 cervical cells and its effects on the phosphatidylinositol 3-kinase (PIK3)/p-Akt/p-phosphatase and tensin homolog (PTEN) signaling pathway. Both parental and paclitaxel-resistant (PTX-R) HeLa cells were exposed to either BYL-719 (BYL, 1.56–100 µM) or LY294002 (LY, 1.56–100 µM) alone or in combination with a fixed molar ratio of PTX (PTX:BYL=1:5, PTX:LY=1:5 ), while parental ME180 and PTX-R ME180 cells were exposed to either BYL (0.078–5 µM) or LY294002 (1.56–100 µM) alone or in combination with PTX at a fixed molar ratio (PTX:BYL=4:1, PTX:LY =1:2.5). The dose–effect curves for PTX alone (green), PTX alone (blue) and combination (red) are shown in (**A**) and (**C**) panels for parental and PTX-R HeLa/ME180 cells, respectively. A multiple effect–level isobologram analysis is shown in (**B**) and (**D**) panels for a 90% effective dose (ED90) (⊙), ED75 (+) and ED50 (х) in both parental and PTX-R HeLa/ME180 cells. Experimental data points, represented by dots located below, on, or above the line, indicate synergism, additivity, and antagonism, respectively. (**E**) The combination index (CI) values for BYL or LY in combination with paclitaxel at ED50, ED75, ED90, ED95 and ED50 -95 (average of CI values at ED50, ED75, ED90, and ED95) for parental and paclitaxel-resistant HeLa and ME180 cell lines. (**F**) PI3K-110α, p-AKT^S473^, p-PTEN, p–3-phosphoinositide-dependent protein kinase 1 (PDK1), and p–glycogen synthase kinase (GSK)-3β were analyzed by western blots in parental and paclitaxel-resistant HeLa and ME180 cell lines after 48 h treatment with BYL-719 (BYL) or LY294002 (LY) and paclitaxel, either alone or in combination. GAPDH was used as an internal control.

To test the combined effect of BYL or LY plus paclitaxel by MTT assay, fixed drug ratios were selected on the basis of each single drug concentration that led to a 50% growth inhibition in parental HeLa and ME180 cells (Table 1). A combination index (CI) was used after 48 h treatment with each reagent alone or in combination. CI values were calculated using CalcuSyn software (version 2.11; Biosoft, Cambridge, UK), at fraction affected (Fa) values of 0.50, 0.75, 0.90, and 0.95, meanwhile ED50-95 was calculated with average value of ED50, ED75, ED90 and ED95 as previously reported [31]. The representative effect curves and isobolograms were drawn in Figure 3A–D. A synergistic effect was found in half the cell population when treating parental HeLa and PTX-R HeLa cells with a combination of PTX and BYL (ratio at 1:5), while stronger synergistic effects at ED75, ED90, and ED95 were noted compared to the effects at ED50 when treating parental HeLa and PTX-R HeLa cells with a combination of PTX and LY (ratio at 1:5; Figure 3E). More enhanced synergistic effects at ED75, ED90 and ED95 were exhibited by parental ME180 and derived paclitaxel-resistant cells when adding a combination of PTX and BYL (ratio at 4:1; Figure 3E) or a combination of PTX and LY (ratio at 1:2.5; Figure 3E) than those at ED50. Generally, as shown in Figure 3E, a synergistic effect (CI < 0.9) under the dose at ED50-95 was observed in both PTX-R HeLa and PTX-R ME180 cells when treated with either BYL or LY in combination with paclitaxel. The synergistic effect, to some extent, varies in different cells due to varying sensitivities to different compounds under an accumulated dose exposure. Based on the CI for experimental values data generated by CalcuSyn, the concentration at each Fa value ≥0.5 with a synergistic effect and a lower concentration than the IC50 of each single reagent alone was selected for further experimental combinations. As shown in Table 1, doses of PTX combined with BYL for both parental HeLa and PTX-R HeLa cells, and parental ME180 and PTX-R ME180 cells were 2 nM:10 μM, 4 nM:20 μM, 5 nM:1.25 μM, and 10 nM:2.5 μM, respectively, showing a strong synergistic effect (except for parental ME180 cells, where a slight synergism was shown). The treatment of parental and PTX-R HeLa cells with PTX combined with LY at 2 nM:10 μM and 4 nM:20 μM showed a strong synergistic effect, while in the parental cell lines, ME180 and PTX-R ME180, combination concentrations were 5 nM:12.5 μM and 10 nM:25 μM, with slight synergism noted. This synergistic effect remained for 72h at least after drugs were washed out after an intervention with combination therapy was conducted at 48h (data not shown) [32].

**Figure 4:**
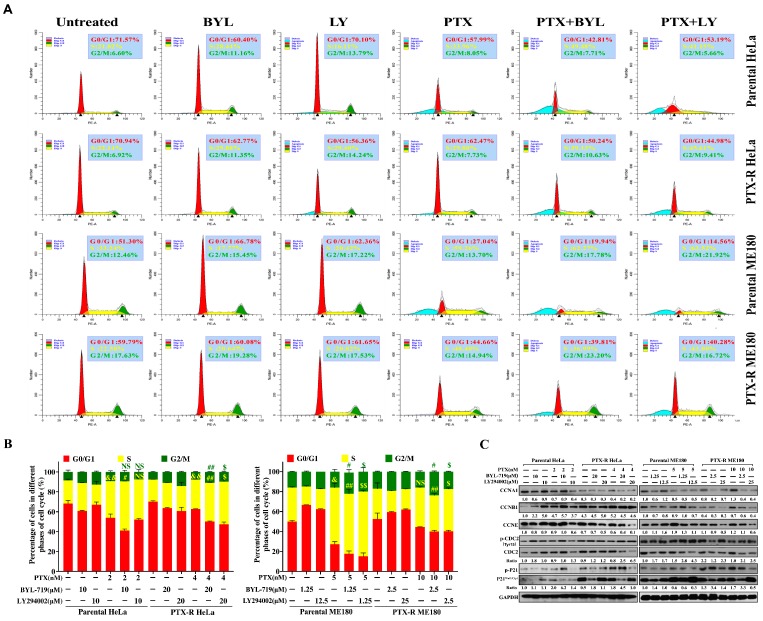
The combination BYL-719 or LY294002 with a low dose of paclitaxel arrests the cell-cycle in S-G_2_M phases in parental and paclitaxel-resistant HeLa and ME180 cells following 48 h treatment. The representative images (**A**) and column graphs (**B**) show that BYL-719 or LY294002 significantly enhanced low-dose paclitaxel–induced S phase arrest in both parental and paclitaxel-resistant HeLa and ME180 cells. Statistical evaluations were performed using Tukey’s one-way ANOVA to obtain significant differences, with normalization based on the untreated group. “&” and “&&” represent *p* < 0.05 and *p* < 0.01, untreated versus paclitaxel in S and G_2_M phase, respectively. “#” and “##” represent *p* < 0.05 and *p* < 0.01, paclitaxel vs. paclitaxel+BYL-719, in S and G_2_M phases, respectively. “$” and “$$” represent *p* < 0.05 and *p* < 0.01, paclitaxel vs. paclitaxel+LY294002, in S and G_2_M phases, respectively. Red, yellow, green, and blue indicate G_0_/G_1_, S, G_2_M, and sub-G_1_ phases (apoptotic cells), respectively. (**C**) The representative western blots (the mean value is presented below the bands from three independent experiments) showing that a combination of BYL-719 or LY294002 with a low dose of paclitaxel caused a significant reduction in cyclin A1, cyclin B1, and cyclin E levels, and induced p-Cdc2/Cdc2 expression. The phosphorylation of p21 in combination-treated groups was enhanced for BYL-719 and paclitaxel, while it was restored for LY294002 and paclitaxel compared to each drug alone. The quantification of bands was performed by densitometric analysis. The ratio values in a western blot (WB) panel represent the ratio of phosphorylated and total proteins, while others are compared with GAPDH.

Further, to confirm whether the PI3K pathway was blocked when using PI3K inhibitors in the paclitaxel combination, western blotting was conducted. PI3K-110α, p-phosphatase and tensin homolog (PTEN), and p–3-phosphoinositide–dependent protein kinase 1 (PDK1) expression were not downregulated in parental HeLa cells when treated with 2 nM PTX +10 μM BYL for 48 h. PI3K-110α/p-PTEN/p-PDK1/p-glycogen synthase kinase 3 (GSK)-3β was not inhibited by combined PTX (5 nM) and LY (10 μM) in parental ME180 cells. However, a synergistic blocking of the PI3K-110α/p-PTEN/p-PDK1/p-GSK-3βsignaling pathway occurred as well as the decreased expression of PTX-induced p-AKT^Ser473^ activation in PTX-HeLa and PTX-R ME180 cells when BYL or LY were combined with PTX, compared to each reagent alone. The same synergistic p-AKT^Ser473^ inhibitory effect was exerted on parental HeLa and ME180 cells after combined PTX and BYL or LY. It was also found that p-AKT^Ser473^/p–GSK-3β was a more effective response marker when treating with PTX and BYL instead of LY in PTX-HeLa and PTX-R ME180 cells (Figure 4).

These results suggested that inhibition of the PI3K pathway could synergistically enhance paclitaxel-induced cytotoxicity in paclitaxel-resistant HeLa and ME180 cells. 

### 2.4. PI3K Inhibitors Synergize Paclitaxel-Induced Cell-Cycle S-G_2_M Phase Arrest And Potentiate Pro-Apoptotic Effects in Paclitaxel-Resistant HeLa and ME180 Cells

As it can be difficult to distinguish between cytotoxicity and an absence of proliferation in the MTT assay [34], cell-cycle and cell apoptosis assays by flow cytometry were performed, respectively. When PTX-R HeLa or PTX-R ME180 cells were treated with BYL (or LY) + PTX, a significantly increased percentage of cells in the S–G_2_M phase was found compared to using each reagent alone. The result of a typical experiment is shown (Figure 4A). Paclitaxel induced S arrest in both parental HeLa and PTX-R HeLa cells, as well as parental ME180 cells. After co-treatment with BYL or LY, a significant increase in the number of cells in the S phase was observed in PTX-R HeLa cells (33.96% and 49.48%, Figure 4A), compared to PTX treatment alone (29.80%, ##*p*<0.01, $*p*<0.05, Figure 4B). G_2_M phase arrest was significantly increased by both BYL or LY with PTX combination therapy in PTX-R HeLa cells, but not in parental cells. The G_0_/G_1_ phase for combination-treated PTX-R ME180 cells decreased to 39.81% (P+B) and 40.28% (P+L) in comparison with the PTX only–treated group (44.66%). The number of G_2_M cells with a proportion of 23.20% and 16.72% was significantly increased in PTX-R ME180 cells, compared with the paclitaxel-treated group (14.94%, #*p*<0.05, $*p*<0.05, Figure 4B), resulting in S–G_2_M arrest. The enhancement of S–G_2_M arrest was also detected when using a combination of PTX with BYL or LY in parental ME180 cells compared to PTX treatment alone. Further, western blots were used to analyze alterations in the cell-cycle–related proteins, cyclin A1 (CCNA1), cyclin B1 (CCNB1), CCNE, Cdc2, and p21. Typical images are shown in Figure 4C: Cyclin A1, which takes part in the rate-limiting step for DNA replication; cyclin E, which controls cell entry from late G_1_ to S phase; and cyclin B1 and Cdc2 complex, which control cell-cycle progression from the G_2_ to M phase, were reduced with PI3K inhibitor and PTX treatment. A combination of LY, but not BYL, with PTX, induced p21 dephosphorylation that suppressed Cdc2 kinase activation in the G_2_/M transition in both PTX-R HeLa and PTX-R ME180 cells.

**Figure 5:**
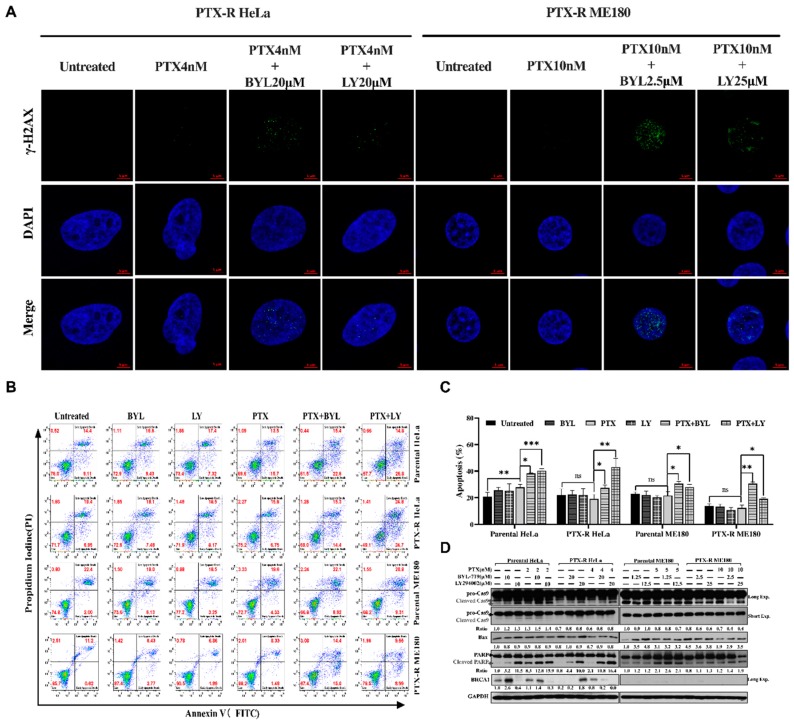
The effect of a combination BYL-719 or LY294002 with a low dose of paclitaxel on DNA damage and cell apoptotic effects on parental and paclitaxel-resistant HeLa and ME180 cells following 48 h treatment. (**A**) The representative images formation of γ-H2AX foci (green) counterstained with DAPI (blue) to distinguish nuclear morphology after paclitaxel treatment alone or in combination with BYL-719 or LY294002 in both PTX-R HeLa and ME180 cell lines at 48h. The scale bar=10 µm. At least 100 cells were counted in each experiment. The cells with ≥5 γ-H2AX discrete foci/nucleus were scored as positive. (**B**) The representative flow cytometry profiles of a comparison of combined BYL-719 (or LY294002) and paclitaxel treatment of parental and paclitaxel-resistant HeLa and ME180 cells as well as column graphs (**C**) showed that BYL-719 or LY294002 significantly potentiated the pro-apoptotic effects of paclitaxel in both parental and paclitaxel-resistant HeLa and ME180 cells compared to each drug alone. The data are presented as the mean  ±  standard deviation (SD) of three experiments; *p < 0.05; **p < 0.01; ***p < 0.001. (**D**) The representative bands (the mean value from three independent experiments is presented below the bands) related to the apoptosis markers, cleaved-poly-(ADP -ribose) polymerase (PARP), Bax, and cleaved caspase-9, and BRCA1 were detected by western blot (WB) after the exposure of cells to BYL-719 or LY294002 and paclitaxel, either alone or in combination, for 48 h. The ratio values in the WB panel represent the ratio of phosphorylated and total proteins. Others use GAPDH as a loading control.

To explore the effects of PI3K inhibitor and PTX on apoptosis in paclitaxel-resistant cells, apoptosis assays were performed by flow cytometry. As shown in Figure 5D, the apoptotic cell population was significantly increased after both parental, PTX-R HeLa and ME180 cells were co-treated with PTX and BYL or LY compared to untreated and PTX-treated groups (*p < 0.05, **p < 0.01, ***p < 0.01, Figure 5E). To investigate the regulatory mechanism of the sensitization of BYL or LY on PTX-R HeLa and PTX-R ME180 cells to paclitaxel, the expression of pro-apoptosis family proteins, such as Bax, cleaved caspase-9 and cleaved poly ADP ribose polymerase (PARP), in PTX-R ME180 and PTX-R HeLa cells was detected by western blot. As shown in Figure 5F, Bax, cleaved caspase-9 and cleaved PARP were markedly activated after PTX-R HeLa cells were treated with a combination of a PI3K inhibitor and paclitaxel rather than treated with paclitaxel alone. Based on these observations, the authors concluded that the PI3K inhibitor could enhance the anti-proliferative activity of paclitaxel in paclitaxel-resistant HeLa and ME180 cells by blocking cell-cycle progression and increasing apoptosis.

### 2.5. PI3K Inhibitors Synergitically Potentiate Paclitaxel Induced DNA Damage and Pro-Apoptotic Effects in Paclitaxel-Resistant HeLa and ME180 Cells

It is commonly thought that S phase arrest is associated with DNA replication and synthesis to promote cell-cycle transition. However, there were unexpected results which showed that both PTX-R HeLa and PTX-R ME180 cells were arrested at S phase after combination treatment according to our flow cytometric data (Figure 4). The authors wondered whether, rather than entering into S-phase, arrest in this phase was controlled by checkpoint proteins that were meant to slow down the cell cycle in order for the cells to overcome the applied replication stress (provoking replication block and/or DNA damage) [35]. To address this question, a well-known DNA damage marker, γ-H2AX was monitored using confocal laser scanning microscopy (CLSM) to further clarify what the cells experienced during S-G_2_M phase arrest. A flow cytometric assay was also utilized to evaluate the apoptotic status after combination treatment. As shown in Figure 5A, for both PTX-R HeLa and PTX-R ME180 cell lines examined, a remarkable increase in green dots which are distinguishable from apoptotic bodies(Figure S1A), indicating elevation of γH2AX, was seen after exposure to paclitaxel in combination with BYL-719 or LY294002 than paclitaxel alone or the untreated group (Figure 5A and Figure S1B,*p < 0.01, **p < 0.01). This led to DNA replication fork arrest and exacerbated DNA damage, which ultimately resulted in cell apoptosis (Figure S1B). The apoptotic cell population was significantly increased after both parental, PTX-R HeLa and ME180 cells were co-treated with PTX and BYL or LY compared to the untreated and PTX-treated groups (*p < 0.05, **p < 0.01, ***p < 0.01, Figure 5B,C). To investigate the regulatory mechanism of the sensitization of PTX-R HeLa and PTX-R ME180 cells by BYL or LY to paclitaxel, the expression of pro-apoptosis family proteins, such as Bax, cleaved caspase-9 and cleaved poly-(ADP -ribose) polymerase (PARP), in PTX-R ME180 and PTX-R HeLa cells was detected by western blot. As shown in Figure 5D, Bax, cleaved caspase-9 and cleaved PARP were markedly activated after PTX-R HeLa cells were treated with a combination of a PI3K inhibitor and paclitaxel rather than treated with paclitaxel alone. It is noted that BRCA1 was significantly deregulated in combination treatment groups compared to paclitaxel treatment alone or the untreated groups (Figure 5D). This may indicate that loss of BRCA1 leads to tumor cells undergoing replication stress with inefficient DNA [36]. Based on these observations, it was concluded that the PI3K inhibitor could enhance the paclitaxel-mediated mitotic block as well as enable cells bear more DNA replication stress, leading to DNA damage and apoptosis in paclitaxel-resistant HeLa and ME180 cells.

### 2.6. Combination of Paclitaxel with PI3K Inhibitors Decreased Paclitaxel-Resistant HeLa and ME180 Cell Migration and Invasion

**Figure 6:**
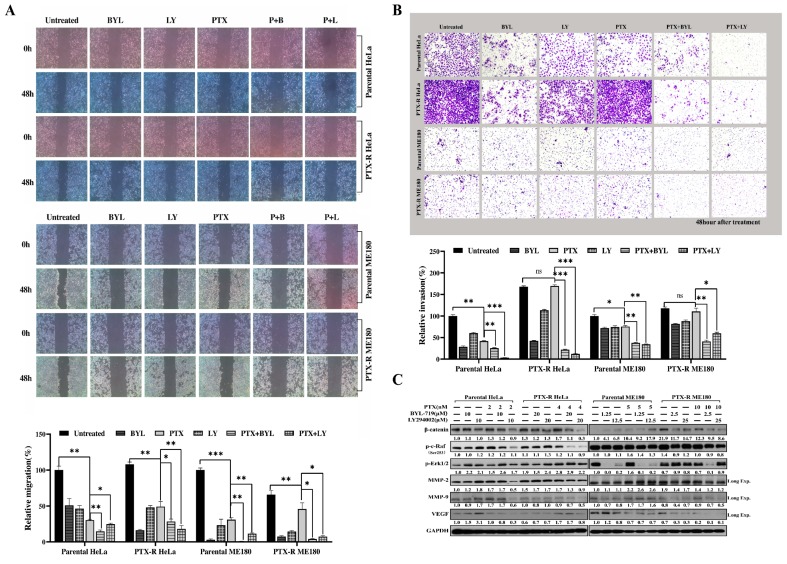
The effects of combined BYL-719 or LY294002 and paclitaxel on migration and invasion in parental and paclitaxel-resistant HeLa and ME180 cells after 48 h. (**A**) Cell migration was observed using a wounding healing migration assay that was quantified by Image J, measuring the wound area at both 0 h and 48 h time points with a mean value of four different fields per scratch. The data are representative images of three independent experiments. Original magnification, ×100. Column graphs for the quantification of cell migration after various treatments is presented as the mean ± standard deviation (SD); *p < 0.05; **p < 0.01; ***p < 0.001. (**B**) Cell invasion was determined using a transwell invasion assay. Original magnification, ×200.Quantification of the number of invading cells is presented as the mean ± SD of three experiments; *p < 0.05; **p < 0.01; ***p < 0.001. (**C**) The representative western blots (mean value is presented below the bands from three independent experiments) of migration and invasion molecular pathways related to β-catenin, p–c-raf, p-extracellular signal regulated kinase (ERK)1/2, metalloproteinase (MMP)-9, MMP-2 and vascular endothelial growth factor (VEGF) expression after treatment with a combination of BYL-719 (or LY294002) with paclitaxel or paclitaxel alone in parental and paclitaxel-resistant HeLa and ME180 cells for 48 h.

Tumor migration and invasion are major obstacles for successful chemotherapy. To evaluate the anti-metastatic potential of PI3K inhibitors in cervical cancer, the authors performed a wound healing assay to measure the speed of cell migration. The representative microscopic images taken at ×100 magnification are shown in Figure 6A. It was found that compared with either the untreated control or paclitaxel alone–treated group, paclitaxel combined with a PI3K inhibitor significantly decreased the speed of migration. The authors also found that the combination of a PI3K inhibitor with paclitaxel decreased cells invading through Matrigel and migrating through the membrane into the bottom chamber (Figure 6B). This indicated that paclitaxel combined with a PI3K inhibitor could reduce the invasion and migration by the two paclitaxel-resistant cervical cancer cell lines rather than paclitaxel treatment alone (*p < 0.05; **p < 0.01; ***p < 0.01; Figure 6A,B). Additionally, our data showed that BYL or LY combined with PTX synergistically enhanced PTX anti-metastatic ability in both parental HeLa and ME180 cell lines (*p < 0.05; **p < 0.01; ***p < 0.01; Figure 6A,B). Further, western blotting revealed that expression of the epithelial–mesenchymal transition–related proteins, β-catenin, p–c-raf, and matrix metalloproteinase (MMP)-2, was highly elevated in paclitaxel-resistant cells (Figure 6C). MMP-2/9 and vascular endothelial cell growth factor (VEGF; angiogenesis-related factor) were abolished when treating cells with BYL (or LY) and PTX for both PTX-R HeLa and PTX-R ME180 cell lines compared to paclitaxel alone. No significant inhibition of p-ERK1/2 was observed in either BYL and PTX–treated PTX-R HeLa cells, nor in LY and PTX–treated PTX-R ME180 cells. Taken together, these results indicated that a combination of PTX with PI3K inhibitors does not only decrease migration ability, but also the invasive properties of paclitaxel-resistant HeLa and ME180 cells.

## 3. Discussion

In this study, the authors attempted to characterize paclitaxel resistance in association with the PI3K pathway by performing apoptosis and migration/invasion assays in cervical cancer cell lines. First, TCGA and GEO databases were used to illustrate the important role of *PI3KCA* in cervical carcinoma. Next, paclitaxel-resistant cervical cancer cell lines (PTX-R ME180 and PTX-R HeLa) were established. This study observed that paclitaxel resistance was acquired through PI3K activation. Third, it was found that treating paclitaxel-resistant cervical cancer cell lines with a combination of paclitaxel and PI3K inhibitors led to anti-tumorigenic effects, such as arresting the cell cycle in the S–G_2_M phase, inducing accumulation of γ-H2AX foci to respond DNA damage and apoptosis and causing decreased migration/invasion. Altogether, our data suggested that a combination of paclitaxel and a PI3K inhibitor can overcome paclitaxel resistance in cervical cancer.

Signaling through the PI3K/Akt pathway in cancer contributes to a tumorigenic phenotype through effects on multiple cellular processes. The induction of mutations and/or the amplification of *PIK3CA* are frequent events found in advanced cervical cancers [37] (Figure 1C,F). Paclitaxel can activate Akt, a serine/threonine protein kinase and a downstream target of PI3K. This was consistent with our finding of the increased activation of PI3K-110α/p-PTEN/p-Akt^Ser473^/p–c-raf (Figure 3E,F; Figure 4 and Figure 6C, 1st Lane versus 4th lane versus 7th lane) during the acquisition of paclitaxel resistance. This may indicate that activation of the PI3K pathway is involved in the progression and prognosis of multiple cancers [38,39]. Enhanced downregulation of the phosphorylation-induced activation of Akt, PTEN, and PDK1was also observed in combination with a PI3K inhibitor, showing a synergistic anti-tumor efficacy compared to paclitaxel alone in paclitaxel resistance cells (Figure 3). Notably, p-GSK-3β was a sensitive marker when treating PTX-R cells with PI3K inhibitor +PTX combination. Dephosphorylation GSK-3β was observed showing a consistency in that the induction of GSK-3β activity may lead to a reduction tumorigenicity as previously described [40].

Several studies have reported that cell replication could be inhibited by paclitaxel by blocking progression beyond the late G_2_ and/or M phases of the cell cycle in various types of cancer [41,42]. Inhibition of Cdc2/cyclin B1 kinase activity in mammalian cells has been shown to result in G_2_ phase arrest [43]. Consistent with this notion in the present study, it was demonstrated that PI3K inhibitor and paclitaxel combination therapy hampered cell-cycle progression by arresting cells in G_2_–M phases in both PTX-R cell lines through the inhibition of CCNB1 and cdc2 (Figure 4A–C). However, it was unexpectedly shown that the cell cycle was also arrested in S phase (Figure 4A). One of the reasons might be due to DNA repair deficiencies and DNA damage acceleration rather than DNA synthesis with the depression of BRCA1 expression (Figure 5D) and the accumulation of γ-H2AX foci in nuclear (Figure 5A, Figure S1) when using PI3K inhibitor and paclitaxel combination therapy. This DNA damage has been reported in several studies previously [35,44,45]. A recent study revealed that PI3K inhibitor enhanced DNA damage and impaired DNA synthesis during the S phase in mouse models of breast cancer [46]. DNA repair deficiency has also been induced by the S arrest as Ashish Juvekar et al. previously reported [36]. BRCA1-deficient mouse embryonic fibroblasts exhibited a marked time-dependent accumulation of cells arrested in the S phase and a prolonged increase in the G2/M population, followed by extensive cell death. Moreover, Ibrahim et al. highlighted that using a PI3K inhibitor, BKM120 could induce accumulation of γH2AX-positive foci in response to DNA damage in BRCA-proficient triple-negative breast cancer (TNBC) cells. This enables γ-H2AX to be a newcomer and sensitive biomarker to investigate the efficacy of drug combinations in clinical trials. The depressed expression of BRCA1/2 and the increase of poly-ADP-ribosylation mediated by BKM120 could subsequently sensitize PARP inhibition in patient-derived primary tumor xenografts [47]. These findings are consistency with our results, and the addition of PARP inhibitor may be useful for those patients lacking a complete response to a PI3K inhibitor plus paclitaxel combination strategy. However, this remains to be further explored in cervical cancer. Moreover, cyclin A appears in the S phase with the onset of DNA synthesis and associates initially with Cdk2 and later with Cdc2 [48], which are essential for progression from S to G_2_ phases. A decreased cyclin A expression may be a reason for the blockage of the S phase in manganese-treated A549 cells [49] and docosahexaenoic acid-treated Jurkat leukemic cells [50]. The increased phosphorylation (Tyr15) of Cdc2 that coincided with the downregulation of Cdc2 in a feedback loop was also observed in both PTX-R cell lines following PTX and PI3K inhibitor treatment, resulting in the accumulation of DNA damage [51,52]. In addition, it is known that p21^WAF1/CIP1^, which is associated with DNA damage and the cell cycle, inhibits the endogenous inhibitor protein cyclin/CDK complex [53,54]. The authors did not observe an elevated expression of cyclin A1 after paclitaxel resistance was induced in HeLa and ME180 cells (Figure 4C). This has been reported as a chemoresistance-associated biomarker in ovarian cancer [45]. Interestingly, markedly increased p-21 phosphorylation leading to p21^Waf1/Cip1^ inactivation was noted when combining BYL with PTX. However, this needs further investigation.

A previous study showed that a combination of paclitaxel and a mitogen-activated protein kinase inhibitor down-regulated PI3K activity, more so than either agent alone [55]. Moreover, Zhang et al. [56] used a novel dual PI3K/mTOR inhibitor, NVP-BEZ235 (BEZ235), with paclitaxel and observed additive anti-proliferative and pro-apoptotic effects to enhance the nanoparticle albumin–bound (nab)–paclitaxel response in gastric cancer cells. In a recent clinical trial, BYL719 with nab–paclitaxel showed promising evidence for less toxicity and increased anti-tumor efficacy in HER2 negative metastatic breast cancer [57]. Taken together, this supports our data showing that a combination of paclitaxel with targeted therapy may enhance the therapeutic effect to overcome paclitaxel resistance in cervical cancer. Mechanically, cell death by PARP cleavage, activation of caspases and cytochrome c release, which was induced by paclitaxel [58] or additional LY294002 treatment [59], has been reported previously. In this study, it was found that Bax and cleaved PARP were significantly increased when treating PTX-R HeLa and ME180 cells with a PI3K inhibitor and paclitaxel compared with paclitaxel alone. The failure of the induction of caspase-9 activation in both parental ME180 and PTX-R ME180 cells, but not HeLa and its PTX-R sublines, may be due to a higher cellular threshold for the paclitaxel-mediated induction of apoptosis in PTX-R ME180 cells. This indicates that a PI3K inhibitor and paclitaxel combination may initiate the intrinsic pathway, which can be independent of Bax-independent mitochondrial depolarization (Figure 5B–D).

A previous study has shown that VEGF is one of the mediators that transmits PI3K-induced oncogenic signals for tumor growth and angiogenesis [60]. The silencing of PI3K p110alpha greatly decreased VEGF expression, an angiogenesis marker, impeding ovarian tumor growth and angiogenesis by targeting hypoxia-inducible factor 1 alpha in both ovarian cancer cells and tumor tissues [60,61]. In our study, it was found that VEGF was inhibited in cervical cancer cells after treatment with PI3K inhibition and paclitaxel compared to paclitaxel alone. This indicated that inhibiting the PI3K pathway in paclitaxel-resistant cervical cells may overcome tumor metastasis and growth by reducing VEGF and its receptors. A reduction in metastases by the flavonoid, apigenin, was due to its ability to inhibit Akt phosphorylation and subsequently cause a decrease in MMP-9, though not MMP-2, activity [62]. However, a disaffinity was revealed in that when treating ovarian cancer cell lines with a PI3K inhibitor, LY294002, a reduction in gonadotropin-induced MMP-2 activity but with little change in MMP-9 activity was noted [63]. A PI3K/mTOR inhibitor, GSK2126458A, given in combination with dabrafenib efficiently counteracted the stimulating effects of a BRAF inhibitor on invasiveness, and VEGF-A and MMP-9 secretion in A375R melanoma cells resistant to darafenib [4]. In our study, both MMP-2 and MMP-9, as well as β-catenin, and p–c-raf, decreased when a combination of two PI3K inhibitors (BYL-719, LY294002) and PTX was added to PTX-R HeLa and PTX-R ME180 cells, compared to PTX treatment alone (Figure 6C). This indicates that the combination of PI3K inhibitors and PTX showed anti-metastatic potential by suppressing the β-catenin/p–c-raf/MMP-2/MMP-9 axis. Additionally, the lack of efficient inhibition of p-ERK1/2 in PTX-R HeLa cells when treated with BYL and PTX, or in PTX-R ME180 cells when treated with LY and PTX, may due to the use of low doses.

In summary, this study suggests that a PI3K inhibitor has a synergistic effect through multiple pathways when combined with paclitaxel in cervical cancer (graphical abstract). Chemotherapeutic agents, including paclitaxel, generally induce tumor regression through apoptosis, which is modulated through a series of proto-oncogenes and tumor suppressor genes. The relationship between alterations in the PI3K pathway and targeted biologic therapy has also been investigated in metastatic or recurrent cervical carcinoma. However, to the authors’ knowledge, this is the first time that BYL-719 in combination therapy with paclitaxel in paclitaxel resistant cervical cancer in an integrative analysis in vitro has been undertaken. Alterations in the regulation of such apoptotic processes may potentially lead to the increased expression of such PI3K-inducible factors and result in the failure of therapy. A concerted effort to distinguish different PI3K inhibitors in combination with other agents, both in the laboratory and clinic, is also warranted. Additionally, an orthotopic paclitaxel resistant TC-1 tumor model is currently under investigation and the safety and efficacy of this strategy remains to be pursued before clinical trials can be undertaken. In all, although the detailed mechanisms underlying paclitaxel resistance and the PI3K pathway are still under investigation, therapeutic strategies targeting these PI3K activation molecules may represent a new paradigm that restores the chemosensitivity of cervical cancer cells to paclitaxel and provide attractive prospects for the treatment of cervical cancer.

## 4. Materials and Methods 

### 4.1. TCGA and GEO Dataset Analysis

The top 10 candidates for frequency (false discovery rate [FDR] <0.1) of genetic and expression alterations were conducted with an open access database publicly available online (http://www.cbioportal.org) using a Mutation Significance (MutSig) algorithm [64] from 308 patients from The TCGA Cervical Squamous Cell Carcinoma and Endocervical Adenocarcinoma provisional dataset [65,66]. PI3K family member classifications and alterations in cervical cancer were summarized and a tutorial edited using Adobe Illustrator CC software (San Francisco, CA, USA) (https://www.adobe.com/products/illustrator.html).

Raw CEL files from GSE6791, GSE5787, GSE26511, GSE2109, GSE7803, and GSE9750 series were downloaded from the GEO (https://www.ncbi.nlm.nih.gov/gds/; Table S1) repository. A *PIK3CA* gene mRNA expression matrix was quantile normalized and analyzed with GraphPad Prism software (version 8.0.2, San Diego, CA, USA). Further details can be found in the Supplementary Methods.

### 4.2. Cell Lines and Cell Culture

Five human cervical cancer cell lines (HeLa, SiHa, CaSki, C33A and ME180) from the Korean Cell Line Bank (Seoul, Korea) were cultured in Minimum Essential Medium, Dulbecco’s modified Eagle’s Minimum, RPMI-1640 Medium, Eagle’s Minimum Essential Medium, and McCoy’s 5a (Gibco/Thermo Fisher Scientific, Waltham, MA, USA), respectively, with 10% fetal bovine serum (FBS, Gibco) and 1% penicillin–streptomycin (HyClone, Logan, UT, USA) at 37 °C in a humidified atmosphere of 5% CO_2_. The original and *PIK3CA* mutation status, including genomic DNA regions containing mutation sites, of the cells were obtained from supplier data sheets from American Type Culture Collection and the Broad Institute project, Cancer Cell Line Encyclopedia (CCLE) (https://portals.broadinstitute.org/ccle).

### 4.3. Reagents and Primary Antibodies

Two PI3Kα-selective (p110α) inhibitors, BYL-719 (BYL; Alpelisib, #HY-15244) and LY294002 (LY; #HY-10108), were purchased from MedChem Express (Monmouth Junction, NJ, USA) and dissolved in dimethylsulfoxide (DMSO) at 100 mM as a stock solution. Paclitaxel (#T7191) and DMSO were purchased from Sigma–Aldrich (St Louis, MO, USA). Primary antibodies for western blots are listed in Table S2.

### 4.4. Establishment of PTX-Resistant Cell Lines

Paclitaxel-resistant (PTX-R) HeLa, PTX-R CaSki and PTX-R ME180 cell lines were established as Tang et al. have previously described [67]. Parental HeLa and ME180 cells were treated with a dose escalation of paclitaxel from 1 to 20 nM (parental CaSki cells were treated with paclitaxel from 1 to 10 nM) until surviving cells recovered and showed a normal exponential growth rate. Surviving cells were harvested and propagated in drug-free medium. Seven months later, HeLa, CaSki and ME180 cells became stably resistant to paclitaxel under 10, 7, and 20 nM, respectively, and all were passaged in paclitaxel-free medium for at least two more months. The 40th PTX-R passage of ME180, 35th PTX-R passage of CaSki and 156th passage of PTX-R HeLa cells were used in the present study.

### 4.5. Cell Viability Assay

Cells (4000–10,000/100 μL/well) were seeded into 96-well plates and incubated for 24 h to achieve 80% confluency in complete medium. The cells were then exposed to different concentrations of PTX, BYL, LY alone and each of two PI3K inhibitors in combination with PTX for 48 h. Later, 5 mg/mL MTT(Sigma–Aldrich) diluted with complete medium was added to each well and incubated for 2 h. DMSO (150 μL/well) was added to solubilize the formazan and plates shaken for 10 min in the dark. The absorbance was measured at 595 nm using a spectrophotometer. The assays were performed in quadruplicate. IC50 values were calculated based on concentration–effect relationships generated by Graph Pad Prism software (Version 8.0.2). The resistance index (RI) was calculated utilizing the following formula: RI = IC50 of drug resistant cells/IC50 of parental cells.

### 4.6. Assessment of Combination Drug Effects

The cells were seeded at a density of 4000~6500/100 μL/well in 96-well plates and different treatments added after 24 h. PTX was used at concentrations of between 1.25–80 nM, while BYL and LY were used at concentrations of between 1.25–80 μM. The PTX and BYL or LY combination ratio for parental HeLa and PTX-R HeLa cells were fixed at the same molar ratio of 1:5, while for parental ME180 and PTX-R ME180 cells, this was fixed at the molar ratios of 4:1 and 1:2.5, respectively. Cytotoxicity was assessed after a 48-h drug exposure for both parental, and PTX-R HeLa and ME180 cells using an MTT assay as described previously. The combined effects of PTX with each of two PI3K inhibitors were analyzed using CalcuSyn software (Version 2.1, Biosoft, Cambridge, UK), based on the median effect principle given by Chou and Talalay(32). CI< 1, = 1 and > 1 demonstrate synergism, an additive effect, and antagonism, respectively.

### 4.7. Semi-Quantitative Reverse Transcriptase-Polymerase Chain Reaction

Total RNA was extracted with TRIzol reagent according to the manufacturer’s instructions. Total RNA (1 µg) in the final 20 µL cDNA system was synthesized by reverse transcription synthesis kit (#04897030001; Roche Diagnostics, Mannheim, Germany). A semi-quantitative reverse transcriptase–polymerase chain reaction (sqRT–PCR) was performed using an AccuPower HotStart PCR PreMix kit according to the manufacturer’s instructions (K-5051; Bioneer, Daejeon, Korea) in a MyGenie 96 Thermal Block (Bioneer, Daejeon, Korea). The cycle sequence of each PCR reaction was performed using an initial denaturation at 94 °C for 5 min, followed by 28 cycles at 94 °C for 30 sec, 52 °C for 30 sec, and 72 °C for 30 sec. After 28 cycles, an additional elongation step was performed at 72 °C for 7 min. PCR products were identified by electrophoresis with 1.0% agarose gels and recorded using a Gel Doc 1000 imaging system (Bio-Rad, Carlsbad, CA, USA). GAPDH RNA served as a housekeeping gene relative to the control.

### 4.8. Apoptosis Analysis

The cells were harvested after 48-h drug treatment and washed twice with ice-cold phosphate-buffered saline (PBS). The cells were then re-suspended in 1× Binding Buffer (1 × 10^6^ cells/mL), stained with Annexin V-FITC and propidium iodide (PI) Apoptosis Detection Kit I (BD Pharmingen, La Jolla, CA, USA) in the dark at room temperature and then analyzed using a BD FACSCanto™ flow cytometry system within 1 h (FACSCanto™ II; Becton Dickinson, Franklin Lakes, NJ, USA). The data was compensated and analyzed by FlowJo software (version 10, Tree Star, Ashland, Oregon, USA).

### 4.9. Cell Cycle (DNA Distribution Analysis)

The cells were seeded at a density of 2.6~4 × 10^5^ cells in 6-cm plates overnight and then starved with serum-free medium for 6 h. After synchronized cell cycling, the cells were exposed to different treatments for another 48 h and collected by trypsinization, washed twice in pre-cold PBS and finally fixed in 1 mL 70% ice-cold absolute alcohol overnight. The cell pellets were washed twice with pre-cold PBS after centrifugation at 2000 rpm for 5 min and then stained with 500 µL FxCycle™ PI/RNase Staining Solution Kit (#1983513, BD Biosciences, San Jose, CA, USA). The cell-cycle distribution was determined using a BD FACSCanto flow cytometry system (FACSCanto™; Becton Dickinson, San Jose, CA, USA). The data was subsequently analyzed with ModFit LT cell-cycle analysis software (Verity Software House, Topsham, ME, USA).

### 4.10. Indirect Immunofluorescence

PTX-R HeLa and PTX-R ME180 cells were grown on glass coverslips in 48 wells at the density of 1~2×10^5^ cells per well for 24 h prior to treatment. After 6h starvation with serum-free medium, the cells were exposed to different treatments for 48 h. The cells were then fixed in 4% paraformaldehyde solution (P2031; Biosesang, Seongnam, Korea) for 20 mins and washed with PBS three times. After 15 mins transparent in 0.1% Triton X-100 and 30 mins blocking in 5% BSA, the cells were washed and incubated with anti-γH2A.X ^Ser139^ (1:100, #9718; Cell Signaling, Danvers, MA, USA) primary antibody in blocking buffer overnight at 4 °C. The next day, the cells were incubated with goat anti-rabbit (1:500, Alexa-488, #111-545-144; Jackson ImmunoResearch, West Grove, PA, USA) secondary antibody conjugated for 1h at room temperature. The coverslips were stained with DAPI in VECTASHIELD Mounting Medium (H-1200; Vector Laboratories, Burlingame, CA, USA) for 10 min at RT. The three-dimensional distribution of γ-H2AX foci images were captured under a 63x magnification objective with oil immersion and analyzed with a Zeiss LSM 800 confocal laser scanning microscope with ZEN 2.3 software (Carl Zeiss, Jena, Germany). The quantification of γ-H2AX foci was conducted manually with a minimum of 100 cells per sample randomly selected. The cells with ≥5 γ-H2AX foci/nucleus were considered to be γ-H2AX foci-positive as previously described [68,69]. 

### 4.11. Western Blotting

The cells were seeded at a density of 0.7~1.2 × 10^6^ cells per 10-cm petri dish and collected after 48 h drug treatment. Whole-cell protein lysates (25ng) were resolved by SDS–polyacrylamide gel electrophoresis (PAGE), transferred to nitrocellulose membranes, and blocked in 5% non-fat dry milk in tris-buffered saline/0.05% Tween-20. Then, the membranes were incubated with primary antibodies overnight at 4°C, followed by incubation with horse radish peroxidase–coupled secondary mouse antibody (1:5000, #213111-01, GeneTex, Irvine, CA, USA) or rabbit (1:5000, #31460, Invitrogen, Carlsbad, CA, USA) at room temperature. The blots were visualized using enhanced chemiluminescence detection reagents (#198506/1859598, Thermo Scientific, Rockland, IL, USA) and exposed to hyper X-ray film (GE Healthcare, Chicago, IL, USA). The band intensities were analyzed using Image J 1.52 software (National Institutes of Health, Bethesda, MD, USA).

### 4.12. Cell Migration (Wound Healing Assay)

The cells (1.2~2 × 10^5^ per well) were grown in 6-well plates overnight. The monolayer was artificially injured by scratching across the plate with a pipette tip (approximately 1 mm in width) when cell confluence reached 70–80%. The wells were washed with PBS twice to remove detached cells or cell debris and the medium changed to one with or without different drugs. After 48 h, scratched wound areas were photographed under each condition and cell migration was quantitatively analyzed by Image J (NIH, Bethesda, MD, USA) to determine areas (A) of the initial (0 h) and healing scratches (48 h). The migration ratio was calculated using the following formula: Cell migration ability = (1 – A_48h_/A_0h_) × 100%.

### 4.13. Cell Invasion (Transwell Assay)

The cells (0.3 ~1 × 10^4^ per well) in the medium containing low serum (1%) were seeded into 24-well plates by adding the cell suspension to 6.5-mm upper chambers of transwells with 8-mm pores (BD Biosciences, Franklin Lakes, NJ, USA) that had been pre-coated for 2 h with Matrigel (BD Biosciences, Bedford, MA, USA) in a 37 °C incubator. Meanwhile, 0.65 mL of 20% FBS complete medium, with or without different concentrations of compounds, was added to the lower chamber as a chemoattractant. After 48 h, the invading cells that had not migrated through the filter in the transwell inserts (on the upper surface) were removed with a cotton swab, and the cells that had migrated to the lower chamber were fixed in paraformaldehyde and stained with a 0.1% crystal violet solution. Four random fields were selected and photographed under an EVOS XL digital inverted microscope (Life Technologies, Carlsbad, CA, USA) at a magnification of 200×.

### 4.14. Statistical Analysis

The assays were performed in at least three independent experiments. The student’s t test and one-way ANOVA were used to assess the differences between the two groups. All results were analyzed and presented as the mean ± standard deviation (SD) with GraphPad Prism (Version 8.0.2). *p* < 0.05 was considered statistically.

## Figures and Tables

**Table 1:** Experimental CI Values for BYL-719 or LY294002 Combination with Paclitaxel.

Combineation	Cell lines	PTX (nM)	BYL-719 (μM)	LY294002 (μM)	Fa	CI	Mode
PTX+ BYL-719	Parental HeLa	2	10		**0.55**	**0.699**	+++
	PTX-R HeLa	4	20		0.55	0.53	+++
	Parental ME180	5	1.25		0.58	0.856	+
	PTX-R ME180	10	2.5		0.6	0.65	+++
PTX+ LY294002	Parental HeLa	2		10	0.65	0.658	+++
	PTX-R HeLa	4		20	0.7	0.629	+++
	Parental ME180	5		12.5	0.6	0.898	+
	PTX-R ME180	10		25	0.6	0.856	+

^1^The combination index (**CI**) is based on that those described by Chou and Talalay [33]: A quantitative measure of the degree of drug interaction in terms of additive effect (CI = 1), synergism (CI < 1), or antagonism (CI > 1) for a given endpoint of the effect measurement. The ranges of CI and the symbols (+++, ++, +) were defined as synergism, moderate, slight synergism effects based on Chou’s earlier report. **Fa**: the fraction affected by the dose.

## Supplementary Materials

Supplementary materials can be found at https://www.mdpi.com/1422-0067/20/14/3383/s1.

## Abbreviations

PI3K	phosphatidylinositol 3-kinase
PTX	paclitaxel
PTX-R	PTX-resistant
BYL,	BYL-719;
LY	LY294002
CI	combination index
MTT	tetrazolium dye
Fa	fraction affected
sqRT-PCR	semi-quantitative reverse transcriptase–polymerase chain reaction
mean ± SD	mean ± standard deviation
RI	resistance index
EMT	epithelial–mesenchymal transition
PDK1	3-phosphoinositide–dependent protein kinase 1
GSK-3β	glycogen synthase kinase 3 beta
PARP	poly (ADP-ribose) polymerase
VEGF	vascular endothelial growth factor
MMP-2/9	matrix metalloproteinase-2/9
WB	western blot
CCNA1	cyclin A1
CCNB1	cyclin B1
CCNE	cyclin E
Cdk1/Cdc2	cyclin-dependent protein kinase
CLSM	confocal laser scanning microscopy
