# Supplementary material for: Inhibition of Phosphatidylinositol 3-kinase (PI3K) Signaling Synergistically Potentiates Antitumor Efficacy of Paclitaxel and Overcomes Paclitaxel-Mediated Resistance in Cervical Cancer
Source: Int J Mol Sci. 2019 Jul 10;20(14):3383. doi: 10.3390/ijms20143383 (PMC6679163; doi:10.3390/ijms20143383)
Supplement: Supplementary file 1 [file ijms-20-03383-s001.zip › Figure S1.pdf]

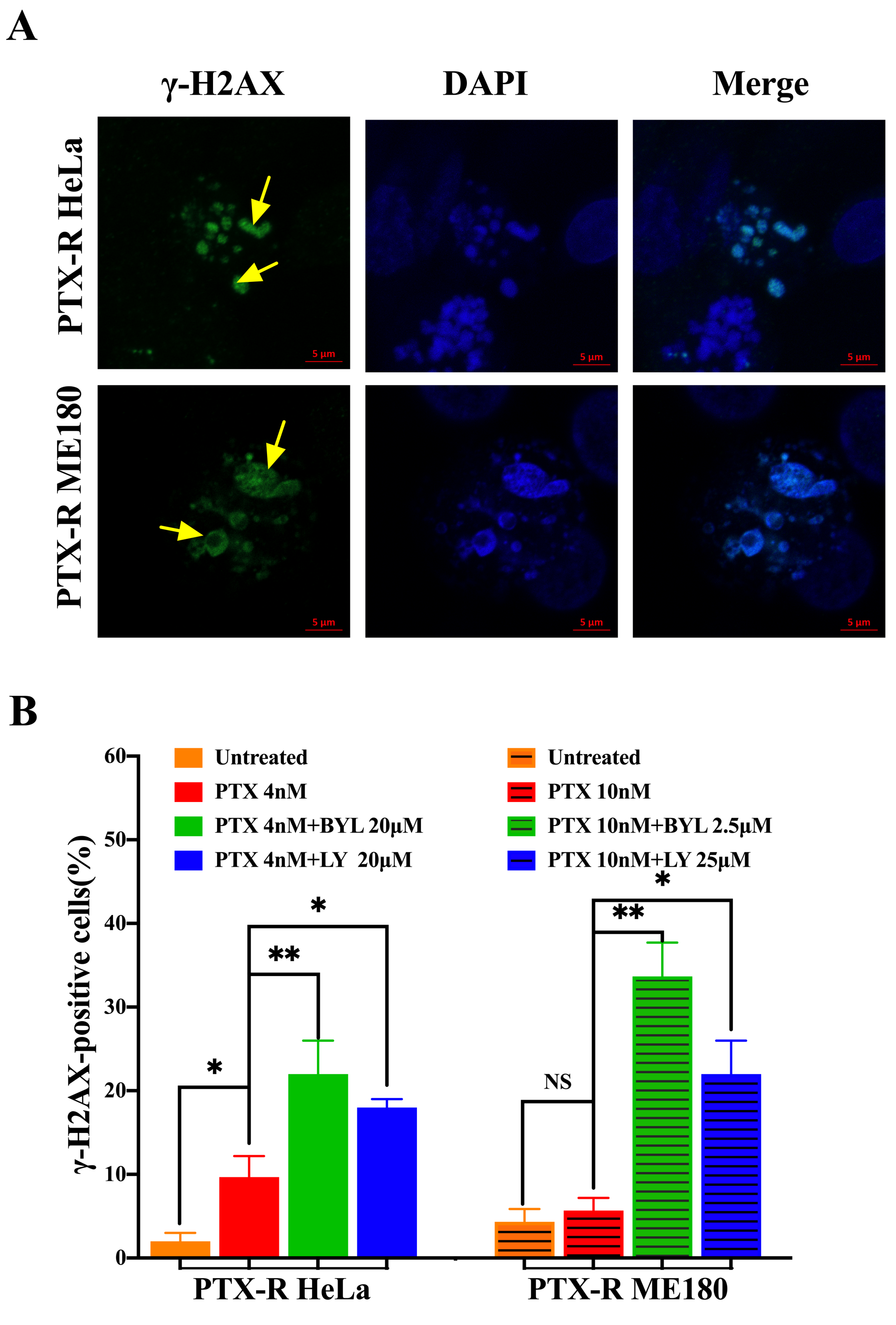

Supplementary Figure S1. (**A**) An intensive cluster staining γH2AX in apoptotic bodies (yellow arrows) which is distinguishable by the entire cell (PMID:22691365). (**B**) Quantification of confocal data as described in **Figure 5A** after paclitaxel treatment alone or in combination with BYL-719 or LY294002 in both PTX-R HeLa and ME180 cell lines at 48h. Cells with ≥5 γ-H2AX discrete foci/nucleus were considered to be positive. Results are presented as the mean ± SD of three independent experiments analyzed by student's t-test. *indicates $P<0.05$, ** indicates $P<0.01$, respectively.
