# Supplementary material for: Inhibition of Phosphatidylinositol 3-kinase (PI3K) Signaling Synergistically Potentiates Antitumor Efficacy of Paclitaxel and Overcomes Paclitaxel-Mediated Resistance in Cervical Cancer
Source: Int J Mol Sci. 2019 Jul 10;20(14):3383. doi: 10.3390/ijms20143383 (PMC6679163; doi:10.3390/ijms20143383)
Supplement: Supplementary file 1 [file ijms-20-03383-s001.zip › Table S1.docx]

**Table.S1** List of GEO Series used in this study

| **Company** | **GEO Accession** | **Platform** | **Normal** | | **Tumor** | | **Reference** | **PMID** |
| --- | --- | --- | --- | --- | --- | --- | --- | --- |
|  |  |  | **Cases** | **Samples** | **Cases** | **Samples** |  |  |
| **Affymetrix** | **GSE6791** | **GPL570** | **8** | **8** | **20** | **20** | **(Pyeon, Newton et al. 2007)** | [**17510386**](https://www.ncbi.nlm.nih.gov/pubmed/17510386) |
|  | **GSE5787** | **GPL570** | **0** | **0** | **16** | **33** | **(Bachtiary, Boutros et al. 2006)** | [**17020965**](https://www.ncbi.nlm.nih.gov/pubmed/17020965) |
|  | **GSE26511** | **GPL570** | **0** | **0** | **39** | **39** | **(Noordhuis, Fehrmann et al. 2011)** | [**21385933**](https://www.ncbi.nlm.nih.gov/pubmed/21385933) |
|  | **GSE2109** | **GPL570** | **0** | **0** | **34+5^a^** | **34+5** | **--** | **--** |
|  | **GSE7803** | **GPL96** | **10** | **10** | **21** | **21** | **(Zhai, Kuick et al. 2007)** | [**17974957**](https://www.ncbi.nlm.nih.gov/pubmed/17974957) |
|  | **GSE9750** | **GPL96** | **24** | **24** | **33+9^b^** | **42** | **(Scotto, Narayan et al. 2008)** | [**18506748**](https://www.ncbi.nlm.nih.gov/pubmed/18506748) |

**Note: a**: metastatic tissue from cervix primary patient; **b**: cell line**.** After the selection of "cervical cancer" as the key word，with "Expression profiling by array" as type and "Homo sapiens" as organism, 42 normal cervix and 194 cervical cancer samples raw CEL files from GPL570 (GSE6791, GSE5787, GSE26511, GSE2109) and GPL96 (GSE7803, GSE9750) platforms were selected. The same platform data was processed together using an R/Bioconductor package oligo in conjunction with Robust Multichip Average (RMA) normalization for background-calibrated, standardized, and log2-transformed perfect match (PM) values that were preformed to remove local biases across samples in order to enable meaningful testing of differential expression.
