# Supplementary material for: Inhibition of Phosphatidylinositol 3-kinase (PI3K) Signaling Synergistically Potentiates Antitumor Efficacy of Paclitaxel and Overcomes Paclitaxel-Mediated Resistance in Cervical Cancer
Source: Int J Mol Sci. 2019 Jul 10;20(14):3383. doi: 10.3390/ijms20143383 (PMC6679163; doi:10.3390/ijms20143383)
Supplement: Supplementary file 1 [file ijms-20-03383-s001.zip › Table S2.docx]

**Table.S2** List of primary antibodies used in this study

| **Antigen** | **Company** | **Cat No.** | **Dilution** | **Size** |
| --- | --- | --- | --- | --- |
| PI3K-p110α | Cell Signaling Technology | #4249 | 1:1000 | 110 kDa |
| p-PTEN（Ser380） | Cell Signaling Technology | #9551 | 1:1000 | 54kDa |
| p-AKT（Ser473） | GeneTex | GTX128414 | 1:1000 | 56kDa |
| p-PDK1（Ser241） | Cell Signaling Technology | #3438 | 1:1000 | 58~68kDa |
| p-GSK-3β（Ser9） | Cell Signaling Technology | #5558 | 1:1000 | 46kDa |
| Caspase9 | Cell Signaling Technology | #9508 | 1:1000 | 47/37/35kDa |
| Bax | Cell Signaling Technology | #2774 | 1:1000 | 21kDa |
| PARP | Cell Signaling Technology | #9542 | 1:1000 | 89,116kDa |
| CyclineA1 | SantaCruz | sc-56301 | 1:1000 | 65 kDa |
| CyclineB1 | Cell Signaling Technology | #4138 | 1:1000 | 55kDa |
| CyclineE | SantaCruz | sc-377100 | 1:1000 | 53kDa |
| p-CDC2（Tyr15） | Cell Signaling Technology | #9111 | 1:1000 | 34kDa |
| CDC2 | Cell Signaling Technology | #9116 | 1:1000 | 34kDa |
| p-P21 | SantaCruz | sc-377569 | 1:1000 | 21kDa |
| P21^Waf1/Cip1^ | Cell Signaling Technology | #2947 | 1:1000 | 21kDa |
| β-catenin | Abcam | ab6302 | 1:2000 | 94kDa |
| p-c-raf（Ser253） | Cell Signaling Technology | #9421 | 1:1000 | 74kDa |
| p-ERK1/2 | Cell Signaling Technology | #9101 | 1:1000 | 42,44kDa |
| MMP-2 | Abcam | ab97779 | 1:1000 | 74kDa |
| MMP-9 | EMD Millipore | IM37 | 1:500 | 92kDa |
| VEGF | Abcam | ab52917 | 1:1000 | 23kDa |
| BRCA1 | Cell Signaling Technology | #9010 | 1:1000 | 220kDa |
| GAPDH | SantaCruz | sc-32233 | 1:5000 | 37kDa |
